# Therapeutic Effect of an Ursolic Acid-Based Nutraceutical on Neuronal Regeneration after Sciatic Nerve Injury

**DOI:** 10.3390/ijms25020902

**Published:** 2024-01-11

**Authors:** Fortuna Iannuzzo, Annunziata Gaetana Cicatiello, Serena Sagliocchi, Elisabetta Schiano, Annarita Nappi, Caterina Miro, Mariano Stornaiuolo, Adriano Mollica, Gian Carlo Tenore, Monica Dentice, Ettore Novellino

**Affiliations:** 1 Department of Pharmacy, G. d’Annunzio University of Chieti-Pescara, 66100 Chieti, Italy; fortuna.iannuzzo@unich.it (F.I.); a.mollica@unich.it (A.M.); 2 Department of Clinical Medicine and Surgery, University of Naples Federico II, 80131 Napoli, Italy; annunziatagaetana.cicatiello2@unina.it (A.G.C.); serena.sagliocchi@unina.it (S.S.); annarita.nappi@unina.it (A.N.); caterina.miro@unina.it (C.M.); 3 Healthcare Food Research Center, Inventia Biotech s.r.l., S. S. Sannitica, 81020 Caserta, Italy; elisabettaschiano@inventiabiotech.com (E.S.); ettore.novellino@unicatt.it (E.N.); 4 Department of Pharmacy, University of Naples Federico II, Via Domenico Montesano 59, 80131 Napoli, Italy; mariano.stornaiuolo@unina.it (M.S.); giancarlo.tenore@unina.it (G.C.T.); 5 Faculty of Medicine and Surgery, Catholic University of the Sacred Heart, 00168 Roma, Italy

**Keywords:** peripheral nerve injuries, ursolic acid, neuronal regeneration, grape pomace, neuromuscular junction, oleolyte, sciatic nerve injury, muscle disorders, muscle atrophy

## Abstract

Peripheral nerve injuries lead to severe functional impairments and long recovery times, with limited effectiveness and accessibility of current treatments. This has increased interest in natural bioactive compounds, such as ursolic acid (UA). Our study evaluated the effect of an oleolyte rich in UA from white grape pomace (WGPO) on neuronal regeneration in mice with induced sciatic nerve resection, administered concurrently with the induced damage (the WGPO group) and 10 days prior (the PRE-WGPO group). The experiment was monitored at two-time points (4 and 10 days) after injury. After 10 days, the WGPO group demonstrated a reduction in muscle atrophy, evidenced by an increased number and diameter of muscle fibers and a decreased Atrogin-1 and Murf-1 expression relative to the denervated control. It was also observed that 85.7% of neuromuscular junctions (NMJs) were fully innervated, as indicated by the colocalization of α-bungarotoxin and synaptophysin, along with the significant modulation of Oct-6 and S-100. The PRE-WGPO group showed a more beneficial effect on nerve fiber reformation, with a significant increase in myelin protein zero and 95.2% fully innervated NMJs, and a pro-hypertrophic effect in resting non-denervated muscles. Our findings suggest WGPO as a potential treatment for various conditions that require the repair of nerve and muscle injuries.

## 1. Introduction

The peripheral nervous system (PNS) is a network of nerves that connects various parts of the body to the brain and spinal cord. It consists of two main components: one that conveys sensory information to the brain and another that transmits signals from the brain to muscles to control movement [1]. In contrast to the central nervous system, the PNS has a greater capacity for self-regeneration [2]. However, the reduced ability to fulfill damage recovery can lead to significant functional impairment and permanent disability [3]. Peripheral nerve injuries (PNIs) affect over one million people worldwide each year and can be caused by various factors, including trauma, nerve compression, infections, toxins, autoimmune diseases, diabetes, and other medical conditions. Despite meticulous microsurgical repair techniques and the use of pharmacological therapies, a significant percentage of PNIs are associated with poor functional outcomes, inadequate nerve recovery, and a loss of sensory and motor functions. These conditions can also lead to chronic pain, muscle atrophy, and profound weakness [4,5]. Non-surgical approaches to treat PNIs have been explored, but clinical implementation remains a distant objective due to various limitations [1,6]. Complementary and safe strategies for promoting peripheral nerve regeneration may be derived from dietary supplementation. Consequently, recent scientific research has focused on the potential contributions of the bioactive compounds naturally present in plant matrixes and food sources [7,8]. In this field, ursolic acid (UA) plays a crucial role in the repair of damaged neurons after PNI [9,10]. UA is a widely distributed pentacyclic triterpenoid in nature with potential health benefits, including antidiabetic, antitumor, antioxidant, and anti-inflammatory effects. This acid is found in various plant types, including rosemary, grape pomace, apple peels, plums, blueberries, raspberries, and other fruit-bearing plants [11]. Literature studies have highlighted that UA effectively promotes the repair of damaged neurons and neural regeneration. In particular, it has been demonstrated to stimulate muscle growth and reduce atrophy, in addition to exhibiting protective effects in neural tissue [12,13,14,15]. However, one of the main limitations of UA is its poor solubility in water, which leads to significant restrictions in its transport and release within the human body, resulting in reduced bioavailability. Nevertheless, the use of dietary oils to enhance the digestion and absorption of poorly water-soluble molecules has garnered increasing attention from researchers [16,17,18]. In particular, a recent study conducted by Liu et al. demonstrated that the use of rapeseed oil significantly improved the bioaccessibility and bioavailability of UA [19]. However, the available literature studies assess the bioavailability and effect of UA in its pure form, and there is limited research on its efficacy when extracted from plant or food sources. Moreover, in recent decades, there has been a growing interest within the scientific community in the valorization of food waste products of the agri-food industry as alternative raw materials for the development of nutraceuticals and food supplements [20,21]. A notable example in this context is grape pomace, an abundant by-product of the wine industry, renowned for its richness in bioactive compounds with a variety of beneficial effects on human health [22,23]. In light of the above considerations, the main objective of this study was to evaluate the effect of a white grape pomace oleolyte (WGPO) (*Vitis vinifera* L. cultivar Fiano) on neuronal regeneration in mice with induced sciatic nerve rescission. The effects of treatment with WGPO, compared to a control (CTR) group treated with only sunflower oil, were monitored at two different time points after denervation: 4 days and 10 days. At the end of the experiment, the mice were sacrificed to collect muscle samples. Therefore, a histological analysis on muscle sections and an evaluation of the mRNA expression levels of muscle atrophy markers (Atrogin-1 and Murf-1) before and after denervation were conducted. To examine the recovery process and the reformation of neuromuscular junctions (NMJs), an mRNA expression analysis of Schwann cell (SC) markers and immunofluorescence staining for α-bungarotoxin and synaptophysin were carried out.

## 2. Results

### 2.1. Quantification of Ursolic Acid (UA) Using HPLC-DAD Analysis

To determine the UA content in WGPO, the oil was deacidified to reduce the free fatty acid content. Then, the determination of the acidity was performed and is expressed as g of oleic acid equivalents (OAE)/g of the analyzed oil. The results show that, after the oil deacidification process, the acidity of the sunflower oil was reduced from 1.64 g OAE/g in the oil sample to 0.27 g OAE/g in the deacidified oil sample. Subsequently, a previously validated HPLC-DAD method [24] was used to quantify the UA in the ethyl acetate extract obtained from WGPO. In particular, the oleolyte extract had a content UA of 0.5 ± 0.03 mg/mL when macerated at 25 °C for 2 h.

### 2.2. WGPO Dietary Supplementation Reduces Denervation-Induced Muscle Loss

**Figure 1:**
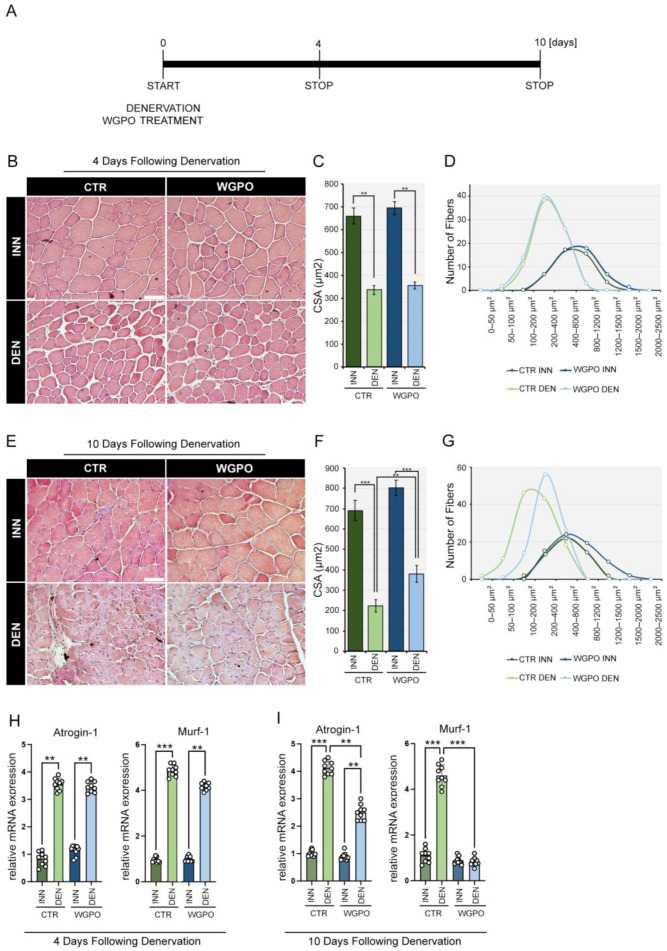
WGPO treatment attenuates skeletal muscle loss after denervation in vivo. (**A**) Schematic representation of the experimental plan. C57/Bl6 wild-type mice treated or not with WGPO were subjected to sciatic nerve cut, and, subsequently, the muscles were collected 4 and 10 days after denervation. (**B**) Hematoxylin & eosin (H&E) staining was performed on tibial anterior (TA) muscle sections of WGPO-treated and control (CTR) mice 4 days after denervation. The denervated muscles were compared to innervated muscles. Magnification 20×, scale bar 50 μm. (**C**,**D**) Mean cross-sectional area (CSA) (**C**) and fiber CSA distribution (**D**) 4 days after denervation. (**E**) H&E staining of TA muscle sections of WGPO-treated and CTR mice 10 days after denervation. Magnification 20×, scale bar 50 μm. (**F**,**G**) Mean CSA (**F**) and fiber CSA distribution (**G**) 10 days after denervation. (**H**) mRNA expression analysis of Atrogin-1 and Murf-1 in denervated gastrocnemius (GC) muscles compared to innervated muscles in WGPO-treated and CTR mice 4 days after denervation. (**I**) mRNA expression analysis of Atrogin-1 and Murf-1 in denervated GC muscles compared to innervated muscles in WGPO-treated and CTR mice 10 days after denervation. Cyclophilin A was used as internal control. Normalized copies of the indicated genes in CTR innervated muscles were set as 1. The data represent the means ± SD from 3 separate experiments with 3 replicates each. Statistical analyses were performed using the two-way ANOVA test and Bonferroni post-test analysis. *n* = 9. ** *p* < 0.01, *** *p* < 0.001.

To test whether WGPO preserves the beneficial effects of pure UA in reducing denervation-induced muscle atrophy, we compared the muscle response to denervation between mice fed a WGPO-enriched diet and mice fed a CTR diet. Denervation was performed via the selective transection of the left sciatic nerve, and the right hindlimb was used as an intrasubject control. In order to evaluate the effects of WPGO at the early post-denervation event and at a later stage of the multiple-step regeneration process after injury, we analyzed the denervated and innervated hindlimb muscles at two different time points (4 days and 10 days) post-denervation (Figure 1A). Both 4 and 10 days post-injury, we observed a statistically significant reduction in the number and diameter of skeletal muscle fibers in the denervated groups compared to the corresponding innervated groups, thereby confirming the effectiveness of the experimental model (Figure 1B–G). Moreover, the results indicate that post-injury treatment with WGPO did not show any statistically significant differences compared to the denervated CTR group in the initial 4 days following denervation (Figure 1B), as highlighted by the analysis of the mean cross-sectional area (CSA) (Figure 1C) and the distribution of the fiber CSA (Figure 1D). In contrast, 10 days after denervation, WGPO significantly reduced the muscle atrophy induced by denervation, resulting in a significant increase in the number and diameter of muscle fibers. Indeed, we observed that, from a histological perspective, the mice treated with WGPO exhibited less pronounced muscular atrophy than the CTR mice. This observation is highlighted by the increased number and diameter of skeletal muscle fibers (Figure 1E). These findings are consistent with the increased average cross-sectional area (CSA) of the fibers (Figure 1F), as well as the broader distribution of the CSA (Figure 1G). Moreover, WGPO dietary supplementation induced trophic changes in “atrophy-related genes”, indicative of the extent of the muscle-wasting condition [25,26]. Therefore, an analysis was conducted on the expression of muscle atrophy markers Atrogin-1 and MurF-1. The results show a statistical increase in the transcriptional upregulation of Atrogin-1 and MuRF1 in both denervated groups. Consistent with previous data, no statistically significant difference was detected after the administration of WGPO 4 days post-injury. However, it is noteworthy that, in the mice treated with WPGO, the increase in the transcriptional upregulation of Atrogin-1 and MuRF1, induced by denervation, was significantly reduced 10 days after the injury compared to in the CTR mice (Figure 1H,I).

### 2.3. Dietary WGPO Promotes Neural Regeneration after Peripheral Nerve Injury

**Figure 2:**
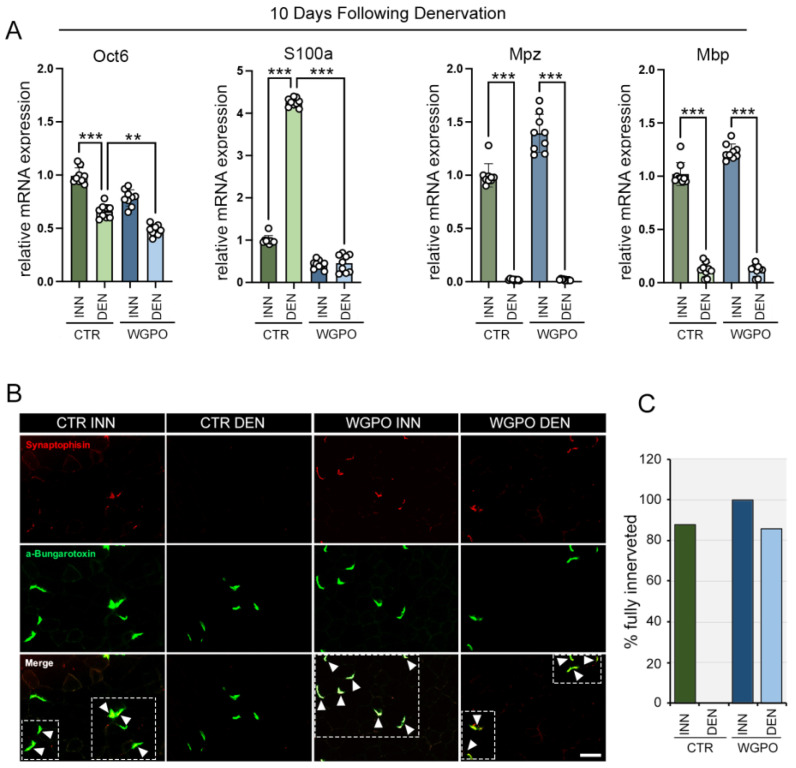
WGPO treatment improves neuromuscular junction regeneration after denervation. (**A**) mRNA expression analysis of neuronal regeneration markers in denervated GC muscles compared to innervated muscles in WGPO-treated and control (CTR) mice 10 days after denervation. Cyclophilin A was used as internal control. Normalized copies of the indicated genes in CTR innervated muscles were set as 1. The data represent the means ± SD from 3 separate experiments with 3 replicates each. Statistical analyses were performed using the two-way ANOVA test and Bonferroni post-test analysis. *n* = 9. ** *p* < 0.01, *** *p* < 0.001. (**B**) Immunofluorescence staining for α–bungarotoxin and synaptophysin in denervated TA muscles compared to innervated muscles of WGPO-treated and CTR mice 10 days after denervation. The white arrows within the dashed rectangles in the merge panels indicate the loci of α-bungarotoxin and synaptophysin colocalization. Magnification 20×, scale bar 50 μm. (**C**) The histograms represent the percentage of fully innervated muscles as in (**B**).

The amelioration of the recovery phase observed in the WGPO-treated mice suggests that WGPO might promote neuromuscular gene expression. To test this hypothesis, we examined the expression profiles of SC-specific markers (Oct6 [27] and S100 [28,29]), myelin basic protein (MBP) [30,31], and myelin protein zero (MPZ) [32,33] in muscles denervated for 10 days compared to innervated muscle in the CTR and WPGO groups. As expected, the mRNA expression analysis showed the downregulation of Oct6, encoding for the transcriptional repressor of myelin-specific genes [34], and a strong induction of S100a, described as an early post-injury response gene [29], in denervated compared to in innervated muscles in the CTR group (Figure 2A). Moreover, MBP and MPZ expression levels were drastically reduced in denervated muscles compared to CTR, indicating a severe myelin loss and the activation of SC proliferation to regenerate the peripheral nerve after injury. Interestingly, WGPO treatment altered the expression of SC differentiation markers, as evidenced by a lower expression of the Oct6 transcriptional factor and a drastic reduction in the early marker S100a in WGPO denervated compared to in untreated denervated muscles (Figure 2A). Moreover, as denervation causes synaptic disruption at the neuromuscular endplate [35], we also compared NMJ changes in the WGPO-treated and CTR mice. Following denervation, the overall synaptic organization was strongly perturbed in the tibial anterior muscles, as shown by the large proportion of degenerated endplates (faintly and dispersedly stained with α-bungarotoxin). However, 10 days after neuronal resection, the WGPO mice showed a higher number of α-bungarotoxin-positive puncta colocalizing with synaptophysin in denervated muscle compared to in CTR denervated muscles (Figure 2B,C and Figure S1). Altogether, these data indicate that WPGO significantly reduces the expression of early SC markers (Oct-6 and S-100) and improves NMJ recovery.

### 2.4. WGPO Induces Skeletal Muscle Hypertrophy Preventing Muscle Loss after Denervation

**Figure 3:**
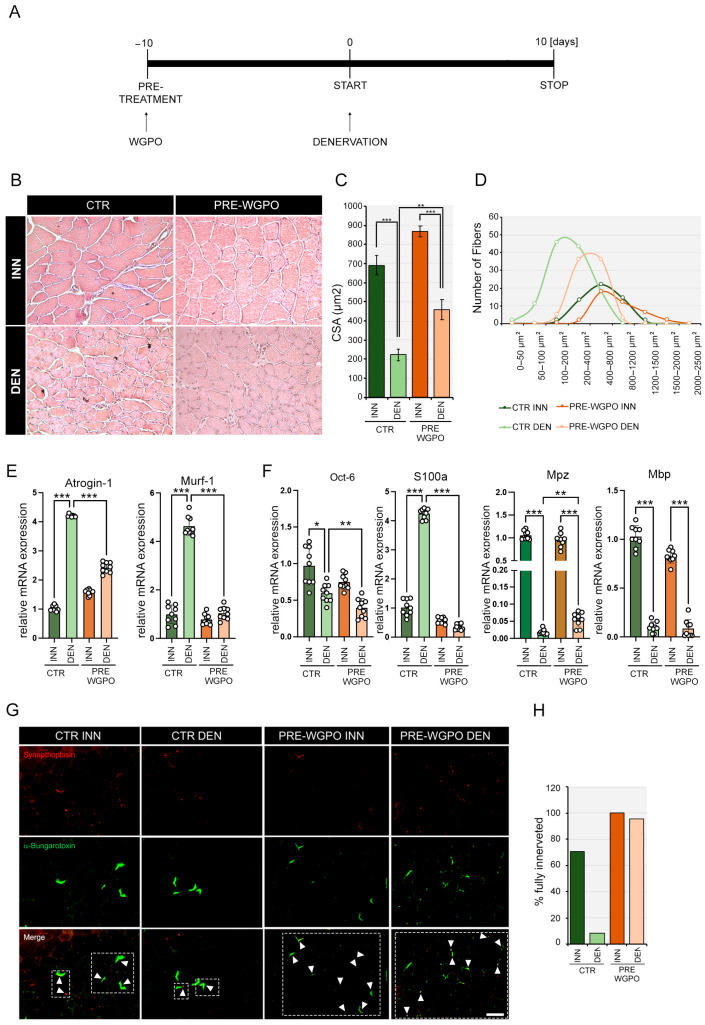
WGPO pre-treatment induces muscle hypertrophy and accelerates the recovery process after injury. (**A**) Schematic representation of the experimental plan. C57/Bl6 wild-type mice were pre-treated with WGPO for 10 days before denervation. Then, the mice were subjected to sciatic nerve cut, and, subsequently, the muscles were collected 10 days after denervation. (**B**) Hematoxylin & eosin staining was performed on tibial anterior (TA) muscle sections of WGPO-pre-treated and control (CTR) mice 10 days after denervation. Magnification 20×, scale bar 50 μm. (**C**,**D**) Mean cross-sectional area (CSA) (**C**) and fiber CSA distribution (**D**) in same muscles as in (**B**). (**E**) mRNA expression analysis of Atrogin-1 and Murf-1 in denervated GC muscles compared to innervated muscles of WGPO-pre-treated and CTR mice at 10 days after denervation. (**F**) mRNA expression analysis of neuronal regeneration markers Oct-6, S100a, Mpz and Mbp in denervated GC muscles compared to innervated muscles of WGPO-pre-treated and CTR mice 10 days after denervation. Cyclophilin A was used as internal control. Normalized copies of the indicated genes in CTR innervated muscles were set as 1. The data represent the means ± SD from 3 separate experiments with 3 replicates each. Statistical analyses were performed using the two-way ANOVA test and Bonferroni post-test analysis. *n* = 9. * *p* < 0.05, ** *p* < 0.01, *** *p* < 0.001. (**G**) Immunofluorescence staining for α–bungarotoxin and synaptophysin in denervated TA muscles compared to innervated muscles of WGPO-pre-treated and CTR mice at 10 days after denervation. The white arrows within the dashed rectangles in the merge panels indicate the loci of α–bungarotoxin and synaptophysin colocalization. Magnification 20×, scale bar 50 μm. (**H**) The histograms represent the percentage of fully innervated muscles as in G.

The finding that WGPO reduced muscle atrophy 10 days post-denervation led us to hypothesize that it might promote muscle hypertrophy in the absence of atrophy-inducing stress. To test this, the mice were allowed ad libitum access to either a CTR or WGPO-enriched diet for 10 days before the sciatic nerve cut (Figure 3A). Compared to the CTR mice, the mice that received WGPO exhibited larger skeletal muscle fibers in innervated muscles, as evidenced by both histological analyses (Figure 3B). This is consistent with the observed increase in the analysis of the mean cross-sectional area (CSA) (Figure 3C) and the distribution of the fiber CSA (Figure 3D). Moreover, when the mice were denervated, the dietary WGPO supplementation exerted a beneficial effect on muscle phenotype. Indeed, at 10 days post-denervation, H&E staining showed that the muscles from the WGPO pre-treated mice appeared to be in a more advanced state of recovery characterized by a larger muscle fiber diameter, from an increase in the mean cross-sectional area (CSA) and the distribution of the fiber CSA. Additionally, the analysis of the muscle atrophy markers Atrogin-1 and Murf-1 revealed a statistically significant reduction following the administration of WGPO after induced damage (Figure 3E). Interestingly, WGPO effectively potentiated SC proliferation and the activation of the regeneration program, as evidenced by a lower Oct6 expression in denervated compared to in untreated denervated muscles. Moreover, the early marker S100a was drastically reduced, thus indicating a more advanced regeneration phase, which is also in line with the doubled expression of the MPZ gene necessary for proper myelination. We next compared NMJ changes in the WGPO-pre-treated and CTR mice (Figure 3G,H). The images show an improvement in endplate regeneration with a higher loci of α-bungarotoxin and synaptophysin colocalization in WGPO-pre-treated muscles after the injury compared to in the CTR mice. These findings provide morphological and molecular evidence demonstrating that pre-treatment with WGPO induces hypertrophy in skeletal muscle fibers and enhances the effect in repairing damaged neurons, thereby promoting neural regeneration following PNI.

## 3. Discussion

Ursolic acid (UA) (3β-hydroxy-urs-12-en-28-oic acid) is a pentacyclic triterpenoid carboxylic molecule widely present in herbs, leaves, flowers, and fruits [36]. This molecule, isolated from various medicinal plants, exists in a free form as oleanolic acid or in a complex form with saponin [37]. In recent years, UA and its derivatives have attracted considerable attention for their numerous beneficial effects, such as antioxidant, anti-cancer, analgesic, anti-inflammatory, and antibacterial properties [11]. Actually, the role of UA in neural regeneration has increasingly gained the attention of neurosurgical scientists. Despite the various treatments available to manage this condition, there is still no optimal treatment for perfect and complete functional recovery [1,6]. Growing evidence has confirmed that plant matrixes and their extracts can stimulate nerve growth factor expression and Schwann cell (SC) proliferation after nerve injury, thereby promoting neural regeneration and functional recovery [5]. Among natural bioactive compounds, UA appears to play a key role in repairing damaged neurons after peripheral nerve injury (PNI). In this context, a great number of studies [15,38,39] showed that the natural compound UA exerts genetic and biochemical effects that improve muscle mass and function. A study by Liu et al. investigated the effect of UA on neural regeneration after sciatic nerve injury in BALB/c mice. The authors demonstrated that UA (tested at doses of 10, 5, and 2.5 mg/kg) significantly increased the muscle mass index in the soleus muscle and the quantity and average diameter of myelinated nerve fibers in the damaged sciatic nerve [12]. More interestingly, a study conducted by Kunkel et al. showed that UA reduced muscle atrophy caused by muscle damage or malnutrition and stimulated muscle hypertrophy in mice by enhancing insulin/IGF-I signaling and inhibiting atrophy-associated mRNA expression in skeletal muscle [15]. Later, Bigford et al. demonstrated that UA enhanced mTOR signaling intermediates, independently inhibiting muscle atrophy and promoting hypertrophy. Regarding the upregulation of catabolic genes induced by spinal cord injury, UA treatment attenuated the loss of IGF-1/mTOR signaling and reduced the gene expression levels of the transcription factor FOXO1, the muscle-restricted E3 ligases Atroginin/MAFbx and Muscle Ring Finger-1 (MuRF-1), and the proteasomal protein PSMD11. Furthermore, UA treatment showed effective functional outcomes, improving muscle function, motor coordination, and strength [38]. These findings suggest that UA could be a promising candidate for the treatment of muscle-wasting conditions, owing to its ability to promote muscle growth and enhance insulin sensitivity in muscle tissue. However, the aforementioned studies have focused exclusively on UA in its pure form, with few studies investigating its efficacy when extracted from food sources and plant matrixes. A critical aspect of the use of UA is its poor water solubility, which affects its bioaccessibility and systemic absorption. In this regard, it is well known that the use of dietary oils can significantly improve the bioavailability of lipophilic molecules, opening new possibilities for its effective employment [16,17,18]. In this context, the main novelty of this work is the investigation of the in vivo effects of white grape pomace oleolyte (WGPO) (*Vitis vinifera* L. cultivar Fiano) as a source of UA with beneficial potential for neuronal regeneration. The incorporation of UA, derived from food sources, in an oil matrix is driven by two primary objectives: The first is to overcome the limit of its poor water solubility, significantly enhancing its bioavailability and effectiveness. The second objective involves the valorization of waste products from the agri-food industry, as they are rich sources of bioactive compounds with significant therapeutic potential for the formulation of nutraceuticals and food supplements. This approach not only aligns with the growing trend towards natural and safe treatments but also offers economic and environmental benefits [40]. The choice of sunflower seed oil was made following Maisto et al., who demonstrated the efficacy of this method for the extraction of UA from plant matrixes [24]. Supporting our strategy, a very recent study performed by Liu et al. showed that the bioavailability of UA in an “oil blend” was 500 times higher than that of standard UA [19] because the first was able to form small micelles containing “soft-interface” UA and bile salts, resulting in a more efficient absorption. To investigate the potential protective effect of WGPO on neuronal regeneration, we conducted an in vivo study using mice with induced sciatic nerve rescission. The treatment was performed on two groups of mice: (1) the WPGO group, where the treatment was administered concurrently with the injury, and (2) the PRE-WGPO group, where a pre-treatment was carried out 10 days before the injury. The results demonstrate that the denervated WGPO group had an improved time of recovery compared to the denervated CTR mice, as demonstrated by an increased muscle fiber diameter and a reduced expression of markers of muscle atrophy, such as MuRF-1 (*p* < 0.001) and Atrogin-1 (*p* < 0.01), 10 days after denervation. Conversely, supplementation with WGPO did not exhibit any statistically significant variations 4 days post-injury, indicating that the effects of WGPO become apparent at a more later stage of the regeneration process. Considering that PNI compromises neuromuscular junctions (NMJs), causing atrophy of the denervated muscles, our evaluation did not limit itself to post-synaptic muscular parameters and included the study of the regenerative capacity of the peripheral nerve in preserving the pre-synaptic end of NMJs. For this reason, two markers involved in the formation and functionality of NMJs, namely, synaptophysin and α-bungarotoxin, were analyzed. Synaptophysin indicates the presence and activity of synaptic vesicles in the presynaptic nerve terminal, while α-bungarotoxin is the postsynaptic component of NMJs, particularly focusing on the acetylcholine receptors in the muscle [41]. The presence of both markers (colocalization) is indicative of the presence of NMJs [42]. We conducted an analysis of the immunofluorescent staining of NMJs, where we observed the number of α-bungarotoxin/synaptophysin colocalizations. Following the induction of damage, the overall synaptic organization was greatly disrupted in the tibialis anterior muscles. However, 10 days post-neuronal resection, the WGPO-treated mice displayed a higher number of α-bungarotoxin-positive loci colocalized with synaptophysin (85.7% were fully innervated) in denervated muscles than in denervated CTR muscles, indicating the formation of functional endplates. The observation of an improvement in the neuronal recovery phase in the mice treated with WGPO stimulated our hypothesis that it might promote neuromuscular gene expression. To this aim, we measured SC markers, which have a dynamic pattern of expression during in vivo regeneration, with early SC markers appearing at the early stages of nerve formation (Oct-6 and S100) and late markers being expressed when NMJs are already formed (MPZ and MBP) [43]. Our findings demonstrate that the use of WGPO treatment concurrently with damage led to a statistically significant decrease in the expression of early SC markers, such as Oct-6 (*p* < 0.01) and S-100 (*p* < 0.0001), compared to denervated CTR. Oct-6 is known to encode a transcriptional repressor of myelin-specific genes. Under normal conditions, this repressor is essential for maintaining the balance of myelin production, preventing the overexpression of myelin genes that could lead to dysfunctions [34]. Therefore, its reduction after nerve injuries may facilitate a greater expression of myelin genes, accelerating the process of re-myelination. S100a is an early response marker to damage and plays a crucial role in the immediate response to injury [27]. Its elevated expression is often an indicator of active or ongoing damage [29]. Therefore, a reduction in S100a following nerve injuries indicates that the acute phase of damage is reducing and that the tissue is entering a phase of regeneration and repair. This suggests that WGPO may positively influence the regeneration of peripheral nerves and muscle repair after injuries. A more beneficial effect of WGPO on muscle regeneration was observed in a pre-treatment experiment, where UA was administered 10 days before denervation. Compared to the WGPO group, the PRE-WGPO group exhibited a greater increase in the number and diameter of skeletal muscle fibers post-injury. In addition, it was interesting to observe that, in resting non-denervated muscles, the WGPO pre-treatment exerted a pro-hypertrophic effect. Moreover, an increase of up to 95.2% in fully innervated sites of α-bungarotoxin–synaptophysin colocalizations was noted, suggesting more effective neuronal regeneration in the pre-treated mice. These data indicate that WGPO enhances the maturation process of nerve fibers during the reinnervation phase, especially when administered preemptively to the onset of injury. This observation is consistent with the analysis of neuromuscular gene expression, revealing that pre-treatment with UA not only preserved improvements in early markers but also brought about significant enhancements in modulating late markers, crucial in the advanced stages of regeneration. Indeed, the PRE-WGPO group showed a statistically significant increase in the levels of myelin protein zero (MPZ) (*p* < 0.01), essential for the reconstruction of the myelin sheath and the restoration of nerve functions following injury. This result is of note considering that SCs are derived early in embryonic development from the neural crest cell through two steps, first developing into a precursor cell and then transforming into immature SCs until birth (Oct6-positive cells) [44]. After birth, when immature SCs differentiate into mature SCs, the expression of the downstream myelin-associated genes MBP and MPZ can be detected [45]. The same expression pattern is also observed in the progression of SC differentiation and myelination during sciatic nerve regeneration [46]. Thus, since WGPO reduces the expression of the early marker Oct6 and augments MPZ gene expression, mainly in the pre-treatment experiments, our results indicate a beneficial pro-differentiating effect of WGPO. Therefore, our research demonstrates the beneficial effects of WGPO in the process of nerve fiber formation after sciatic nerve injuries. This is evidenced by improvements in muscle reinnervation and hypertrophy, as indicated by the increase in the number and diameter of muscle fibers, thus contributing to the mitigation of muscle atrophy. Additionally, the ability of WGPO to modulate the expression of SC markers, leading to an increased number of functional NMJs, highlights its potential role in promoting differentiation and remyelination processes. Notably, pre-treatment with WGPO has shown an enhanced effect on nerve regeneration post-injury, and it also exerts a pro-hypertrophic effect on resting non-denervated muscles.

## 4. Materials and Methods

### 4.1. Reagents

All chemicals, reagents, and standards used were analytical or LC-MS grade reagents. Water was treated in a Milli-Q water purification system (Millipore, Bedford, Burlington, MA, USA) before use. Sunflower oil was purchased at a local market. Ursolic acid (purity ≥ 98.5% HPLC) was purchased from Sigma-Aldrich (Milan, Italy).

### 4.2. Oleolyte Preparation Protocol for In Vivo Experiment

White grape pomace (WGP) (*Vitis vinifera* L. cultivar Fiano) was harvested in October 2022 in Lapio (Avellino, Italy). The matrix was then washed and freeze-dried to obtain a homogeneous powder. To prepare the oleolyte, a given weight of WGP was added to a certain volume of sunflower oil (g/mL) at a ratio of 1:4 with an extraction time of 2 h and an incubation temperature of 25 °C. At the end of the matrix maceration in oil, the mixture was centrifuged at 9000 rpm for 10 min. The oil supernatant was collected and stored protected from light at 4 °C until administration to mice.

### 4.3. Ursolic Acid (UA) Extraction Protocol for HPLC-DAD Analysis

#### 4.3.1. Oil Deacidification Procedure

The oil deacidification procedure was conducted to determine the extraction of terpenic acids from the matrix and to decrease the presence of free fatty acids in order to guarantee a low formation of oxidant products that can damage chromatography columns. The deacidification of the oil was achieved through liquid–liquid extraction using a basic solution. Therefore, 250 mL of a sodium carbonate solution (Na_2_CO_3_ 7.5%, *w*/*v*) was mixed with 250 mL of n-hexane, followed by the addition of 500 mL of sunflower oil [47]. This mixture was mixed on a magnetic stirrer for 10 min, after which the organic phase was isolated through liquid–liquid extraction. To eliminate any residual alkaline solution, the organic phase was washed with 1000 mL of water. After stirring the mixture for an additional 10 min, the organic phase was again separated through liquid–liquid extraction. In the final step, the hexane was removed via vacuum evaporation at 35 °C to obtain a deacidified oil.

#### 4.3.2. Measurement of Acidity in Oil Samples

To determine the free fatty acid content, the acidity of the oil obtained from the deacidification process was determined. The determination of acidity was performed in accordance with Regulation (EU) No 2016/1227. A mixture of diethyl ether and ethanol (50:50 *v*/*v*) was neutralized with a solution of potassium hydroxide 0.1 M, to which 300 µL of an ethanolic solution of phenolphthalein 0.03 M was added. An aliquot of oil (2.5 g) was dissolved in 50 mL of the neutralized solvent mixture. The mixture was titrated via stirring with an aqueous solution of potassium hydroxide 0.1 M until the pH indicator changed color. All determinations were performed in triplicate. The acidity was calculated according to the following formula: (V × c × M)/(10 × m), where V is the volume (mL) of the titrated potassium hydroxide solution, c is the concentration (M) of the titrated potassium hydroxide solution, M is the molar mass in grams per mole of oleic acid (OA) (282 g/mol), and m is the mass (g) of the oil sample.

#### 4.3.3. Protocol for the Extraction of Ursolic Acid (UA) 

A volume of 120 mL of sodium carbonate Na_2_CO_3_ 7.5% (*w*/*v*) solution was added to 60 mL of deacidified WGPO. The mixture was stirred for 10 min, and the aqueous phase was separated via liquid–liquid extraction. The aqueous phase was acidified with 2 N hydrochloride acid at pH = 3, frozen, and lyophilized. To the solid residue, 20 mL of ethyl acetate was added. The mixture was vortexed for 1 min and placed in an ultrasonic bath (Branson Fisher Scientific 150 E Sonic Dismembrator, Marshall Scientific, Hampton, VA, USA) for 10 min. The samples were then shaken on an orbital shaker (Sko-DXL, Argolab, Carpi, Italy) at 600 rpm for 10 min and centrifuged at 9000 rpm for 10 min. The supernatants were collected and stored at 4 °C protected from light. The obtained pellets were re-extracted with 10 mL of ethyl acetate using the same procedure. Finally, the extracts obtained were evaporated to dryness under a light stream of nitrogen, reconstituted in dimethylsulfoxide (DMSO) at a concentration of 30 mg/mL, diluted with acetonitrile at a concentration of 5 mg/mL, and stored at −20 °C until analysis [24].

#### 4.3.4. Ursolic Acid (UA) Quantitative Analysis Using HPLC-DAD

The HPLC-DAD analysis method used was validated following the approach previously detailed in [22]. For the analysis, a Jasco Extrema LC-4000 HPLC system, equipped with an autosampler, a binary solvent pump, and a diode-array detector (DAD), from Jasco Inc., Easton, MD, USA, was utilized. Chromatographic separation was carried out on a Kinetex^®^ C18 column (250 mm × 4.6 mm, 5 µm; Phenomenex, Torrance, CA, USA). The mobile phases were water with 0.1% formic acid (A) and acetonitrile (B). Elution was executed with a specific gradient: initially isocratic at 60% B for 0–3 min, followed by a linear gradient to 90% B from 3–20 min, then isocratic at 90% B for 20–24 min, and then returning to the initial condition. The sample injection volume was set at 20 µL, with the column temperature maintained at 30 °C and a flow rate of 1 mL/min. UA quantification was carried out at a wavelength of 205 nm.

### 4.4. In Vivo Experimental Protocols

#### 4.4.1. Mouse Strains

C57BL/6J mice were obtained from Jackson Laboratory (Bar Harbor, ME, USA). In this study, 12-week-old male littermates were used. The animals were handled according to national and European community guidelines, and protocols were approved by the animal research committee of the University of Naples “Federico II”. The mice were housed in cages containing fresh bedding under controlled conditions, namely, 20–24 °C, 50–60% relative humidity, and a 14:10 light–dark cycle. Food and water were available ad libitum on all days for all animals. All animal procedures were approved by the Institutional Animal Care and Use Committee (IACUC) (protocol n. 354/2019-PR).

#### 4.4.2. Real-Time qRT-PCR

Messenger RNAs were extracted with Trizol reagent (Life Technologies, Monza, Italy). Complementary DNAs were prepared with SuperScript VILO Master Mix (Life Technologies), as indicated by the manufacturer. The cDNAs were amplified via PCR in a CFX Connect Real-Time PCR Detection System (Bio-Rad, Milan, Italy), with the fluorescent double-stranded DNA-binding dye SYBR Green (BioRad). Specific primers for each gene were designed to work under the same cycling conditions (95 °C for 10 min, followed by 40 cycles at 95 °C for 15 s and 60 °C for 1 min), thereby generating products of comparable sizes (about 200 bp for each amplification) [48]. Primer combinations were positioned whenever possible to span an exon–exon junction, and RNA was digested with DNase to avoid genomic DNA interference. The primer sequences are reported in Table S1. The relative amounts of gene expression were calculated using Cyclophilin-A as an internal standard control. All samples were run in triplicate. The results, expressed as N-fold differences in target gene expression, were determined as follows = 2 − DCt target − DCt control) [49].

#### 4.4.3. WGPO Dietary Supplementation and Denervation Experiments 

The 12-week-old C57BL/6 male mice (The Jackson Laboratory, Bar Harbor, ME, USA) were randomly assigned to four groups: the CTR (*n* = 10) and PRE-CTR (*n* = 10) groups, which were fed the usual diet containing only the vehicle (sunflower oil), and the WGPO group (*n* = 20) and PRE-WGPO group (*n* = 20), which were fed the usual diet containing WGPO with a dosage of 2.5 mg UA/kg of body weight. In all mice, the sciatic nerve cut was conducted as previously described [50]. The WGPO group received WGPO dietary supplementation contextually to denervation, while the PRE-WGPO group received WGPO supplementation for 10 days before the rescission of the sciatic nerve. To evaluate the WGPO effect on the muscle nerve regeneration process, the mice of each experimental group were collected at two different time points after denervation: 4 days (*n* = 10) and 10 days (*n* = 10). At the end of the experiment, the mice were sacrificed to collect blood and muscle samples for further testing. The muscles were stored at 80 °C for real-time quantitative PCR and histological analyses. Animal experimental protocols were approved by the Animal Research Committee of the University of Naples “Federico II”.

#### 4.4.4. Antibodies

All primary antibodies used for IHC are listed in Table S2, and the dilution is indicated in the list. The following primary antibodies were used: Anti-α-Bungarotoxin Conjugates (1/500 for IHC) from Invitrogen™ (Carlsbad, CA, USA) and Anti-Synaptophysin (1/500 for IHC) from Abcam (Cambridge, UK).

#### 4.4.5. Histology and Immunostaining

Muscles were dissected and frozen in liquid nitrogen-cooled isopentane; 8 mm muscle cryosections were used for histology analyses. Cryostat sections were stained with hematoxylin & eosin (HE) (Sigma-Aldrich, St. Louis, MO, USA) according to classical methods [51,52]. Briefly, for the H&E analysis, cross-sections were fixed in 4% formaldehyde at room temperature for 15 min and stained with H&E. Fiber size distribution was quantified using ImageJ software 1.52k Wayne Rasband, NIH (Bethesda, MD, USA) (http://imagej.nih.gov/ij, accessed on 30 January 2019) [53]. Up to 6 fields of view were captured from the same location within each muscle. Six hundred myofibers were measured per muscle. The images were acquired using a Leica DMi8 microscope. For post-synaptic α-bungarotoxin and pre-synaptic synaptophysin, sections were blocked with blocking solution (0.5% milk, 10% FBS, 1% BSA) for 1 h at room temperature. They were incubated with the primary antibody overnight at 4 °C and secondary fluorescent antibodies (Invitrogen) for 1 h at room temperature. Then, nuclei were stained with DAPI, and sections were mounted in 80% glycerol. Images were captured using a Leica DMi8 microscope using the Leica Application Suite LAS X Imaging (Leica, Berlin, Germany) for the acquisitions.

### 4.5. Quantification and Statistical Analysis 

The results are shown as the mean ± standard deviation (SD) throughout, as specified in the figure legends. Multiple comparisons were assessed using the two-way ANOVA test and Bonferroni post-test analysis. Relative mRNA levels (in which the control sample was arbitrarily set as 1) are reported as the results of Real-Time qRT-PCR, in which Cyclophilin-A served as a housekeeping gene. The real-time PCR results were corrected using the two-way ANOVA test and Bonferroni post-test analysis. In all experiments, differences were considered significant when the *p*-value (*p*) was less than 0.05. Asterisks indicate significance at * *p* < 0.05, ** *p* < 0.01, and *** *p* < 0.001 throughout. The definitions of the *n*-values are reported in the figure legends.

## 5. Conclusions

Our findings suggest that WGPO could be regarded as a promising candidate for the treatment of conditions that require the repair of nerve and muscle injuries. 

## Data Availability

The data used to support the findings of this study are included in the article.

## Supplementary Materials

The following supporting information can be downloaded at https://www.mdpi.com/article/10.3390/ijms25020902/s1.

## References

[1] Lopes B., Sousa P., Alvites R., Branquinho M., Sousa A.C., Mendonça C., Atayde L.M., Luís A.L., Varejão A.S.P., Maurício A.C. (2022). Peripheral Nerve Injury Treatments and Advances: One Health Perspective. Int. J. Mol. Sci..

[2] Zhang M., Li L., An H., Zhang P., Liu P. (2021). Repair of Peripheral Nerve Injury Using Hydrogels Based on Self-Assembled Peptides. Gels.

[3] Modrak M., Talukder M.A.H., Gurgenashvili K., Noble M., Elfar J.C. (2020). Peripheral Nerve Injury and Myelination: Potential Therapeutic Strategies. J. Neurosci. Res..

[4] Wang M.L., Rivlin M., Graham J.G., Beredjiklian P.K. (2019). Peripheral Nerve Injury, Scarring, and Recovery. Connect. Tissue Res..

[5] Muratori L., Fregnan F., Maurina M., Haastert-Talini K., Ronchi G. (2022). The Potential Benefits of Dietary Polyphenols for Peripheral Nerve Regeneration. Int. J. Mol. Sci..

[6] Hussain G., Wang J., Rasul A., Anwar H., Qasim M., Zafar S., Aziz N., Razzaq A., Hussain R., de Aguilar J.-L.G. (2020). Current Status of Therapeutic Approaches against Peripheral Nerve Injuries: A Detailed Story from Injury to Recovery. Int. J. Biol. Sci..

[7] Durazzo A., Lucarini M., Souto E.B., Cicala C., Caiazzo E., Izzo A.A., Novellino E., Santini A. (2019). Polyphenols: A Concise Overview on the Chemistry, Occurrence, and Human Health. Phytother. Res..

[8] Samtiya M., Aluko R.E., Dhewa T., Moreno-Rojas J.M. (2021). Potential Health Benefits of Plant Food-Derived Bioactive Components: An Overview. Foods.

[9] Gudoityte E., Arandarcikaite O., Mazeikiene I., Bendokas V., Liobikas J. (2021). Ursolic and Oleanolic Acids: Plant Metabolites with Neuroprotective Potential. Int. J. Mol. Sci..

[10] Yow Y.-Y., Goh T.-K., Nyiew K.-Y., Lim L.-W., Phang S.-M., Lim S.-H., Ratnayeke S., Wong K.-H. (2021). Therapeutic Potential of Complementary and Alternative Medicines in Peripheral Nerve Regeneration: A Systematic Review. Cells.

[11] Mlala S., Oyedeji A.O., Gondwe M., Oyedeji O.O. (2019). Ursolic Acid and Its Derivatives as Bioactive Agents. Molecules.

[12] Liu B., Liu Y., Yang G., Xu Z., Chen J. (2013). Ursolic Acid Induces Neural Regeneration after Sciatic Nerve Injury. Neural Regen. Res..

[13] Pemminati S., Gopalakrishna H.N., Varma A., Chakraborty M., Bheemesh V., Ratnakar U.P., Ullal S.D., Shenoy A.K. (2011). Effect of Acute Administration of Ursolic Acid on Haloperidol Induced Catalepsy in Albino Mice. J. Pharm. Res..

[14] Shih Y.-H., Chein Y.-C., Wang J.-Y., Fu Y.-S. (2004). Ursolic Acid Protects Hippocampal Neurons against Kainate-Induced Excitotoxicity in Rats. Neurosci. Lett..

[15] Kunkel S.D., Suneja M., Ebert S.M., Bongers K.S., Fox D.K., Malmberg S.E., Alipour F., Shields R.K., Adams C.M. (2011). mRNA Expression Signatures of Human Skeletal Muscle Atrophy Identify a Natural Compound That Increases Muscle Mass. Cell Metab..

[16] Thakur N., Raigond P., Singh Y., Mishra T., Singh B., Lal M.K., Dutt S. (2020). Recent Updates on Bioaccessibility of Phytonutrients. Trends Food Sci. Technol..

[17] Ojeda-Serna I.E., Rocha-Guzmán N.E., Gallegos-Infante J.A., Cháirez-Ramírez M.H., Rosas-Flores W., Pérez-Martínez J.D., Moreno-Jiménez M.R., González-Laredo R.F. (2019). Water-in-Oil Organogel Based Emulsions as a Tool for Increasing Bioaccessibility and Cell Permeability of Poorly Water-Soluble Nutraceuticals. Food Res. Int..

[18] Huang M., Wang Y., Ahmad M., Ying R., Wang Y., Tan C. (2021). Fabrication of Pickering High Internal Phase Emulsions Stabilized by Pecan Protein/Xanthan Gum for Enhanced Stability and Bioaccessibility of Quercetin. Food Chem..

[19] Liu Y., Xia H., Guo S., Li P., Qin S., Shi M., Zeng C. (2023). Effect and Mechanism of Edible Oil Co-Digestion on the Bioaccessibility and Bioavailability of Ursolic Acid. Food Chem..

[20] Piccolo V., Maisto M., Schiano E., Iannuzzo F., Keivani N., Manuela Rigano M., Santini A., Novellino E., Carlo Tenore G., Summa V. (2024). Phytochemical Investigation and Antioxidant Properties of Unripe Tomato Cultivars (*Solanum Lycopersicum* L.). Food Chem..

[21] Sorrenti V., Burò I., Consoli V., Vanella L. (2023). Recent Advances in Health Benefits of Bioactive Compounds from Food Wastes and By-Products: Biochemical Aspects. Int. J. Mol. Sci..

[22] Baroi A.M., Popitiu M., Fierascu I., Sărdărescu I.-D., Fierascu R.C. (2022). Grapevine Wastes: A Rich Source of Antioxidants and Other Biologically Active Compounds. Antioxidants.

[23] Iannuzzo F., Piccolo V., Novellino E., Schiano E., Salviati E., Summa V., Campiglia P., Tenore G.C., Maisto M. (2022). A Food-Grade Method for Enhancing the Levels of Low Molecular Weight Proanthocyanidins with Potentially High Intestinal Bioavailability. Int. J. Mol. Sci..

[24] Maisto M., Piccolo V., Novellino E., Schiano E., Iannuzzo F., Ciampaglia R., Summa V., Tenore G.C. (2023). Optimization of Ursolic Acid Extraction in Oil from Annurca Apple to Obtain Oleolytes with Potential Cosmeceutical Application. Antioxidants.

[25] Bodine S.C., Latres E., Baumhueter S., Lai V.K.-M., Nunez L., Clarke B.A., Poueymirou W.T., Panaro F.J., Na E., Dharmarajan K. (2001). Identification of Ubiquitin Ligases Required for Skeletal Muscle Atrophy. Science.

[26] Cicatiello A.G., Sagliocchi S., Nappi A., Di Cicco E., Miro C., Murolo M., Stornaiuolo M., Dentice M. (2022). Thyroid Hormone Regulates Glutamine Metabolism and Anaplerotic Fluxes by Inducing Mitochondrial Glutamate Aminotransferase GPT2. Cell Rep..

[27] Ryu E.J., Wang J.Y.T., Le N., Baloh R.H., Gustin J.A., Schmidt R.E., Milbrandt J. (2007). Misexpression of Pou3f1 Results in Peripheral Nerve Hypomyelination and Axonal Loss. J. Neurosci..

[28] Gould V.E., Moll R., Moll I., Lee I., Schwechheimer K., Franke W.W. (1986). The Intermediate Filament Complement of the Spectrum of Nerve Sheath Neoplasms. Lab. Investig..

[29] Chernov A.V., Dolkas J., Hoang K., Angert M., Srikrishna G., Vogl T., Baranovskaya S., Strongin A.Y., Shubayev V.I. (2015). The Calcium-Binding Proteins S100A8 and S100A9 Initiate the Early Inflammatory Program in Injured Peripheral Nerves. J. Biol. Chem..

[30] Bhatheja K., Field J. (2006). Schwann Cells: Origins and Role in Axonal Maintenance and Regeneration. Int. J. Biochem. Cell Biol..

[31] García-Suárez O., Montaño J.A., Esteban I., González-Martínez T., Alvarez-Abad C., López-Arranz E., Cobo J., Vega J.A. (2009). Myelin Basic Protein-positive Nerve Fibres in Human Meissner Corpuscles. J. Anat..

[32] Turner B.J., Ackerley S., Davies K.E., Talbot K. (2010). Dismutase-Competent SOD1 Mutant Accumulation in Myelinating Schwann Cells Is Not Detrimental to Normal or Transgenic ALS Model Mice. Hum. Mol. Genet..

[33] Le N., Nagarajan R., Wang J.Y.T., Araki T., Schmidt R.E., Milbrandt J. (2005). Analysis of Congenital Hypomyelinating Egr2Lo/Lo Nerves Identifies Sox2 as an Inhibitor of Schwann Cell Differentiation and Myelination. Proc. Natl. Acad. Sci. USA.

[34] Svaren J., Meijer D. (2008). The Molecular Machinery of Myelin Gene Transcription in Schwann Cells. Glia.

[35] Carnio S., LoVerso F., Baraibar M.A., Longa E., Khan M.M., Maffei M., Reischl M., Canepari M., Loefler S., Kern H. (2014). Autophagy Impairment in Muscle Induces Neuromuscular Junction Degeneration and Precocious Aging. Cell Rep..

[36] Seo D.Y., Lee S.R., Heo J.-W., No M.-H., Rhee B.D., Ko K.S., Kwak H.-B., Han J. (2018). Ursolic Acid in Health and Disease. Korean J. Physiol. Pharmacol..

[37] López-Hortas L., Pérez-Larrán P., González-Muñoz M.J., Falqué E., Domínguez H. (2018). Recent Developments on the Extraction and Application of Ursolic Acid. A Review. Food Res. Int..

[38] Bigford G.E., Darr A.J., Bracchi-Ricard V.C., Gao H., Nash M.S., Bethea J.R. (2018). Effects of Ursolic Acid on Sub-Lesional Muscle Pathology in a Contusion Model of Spinal Cord Injury. PLoS ONE.

[39] Jung S.H., Ha Y.J., Shim E.K., Choi S.Y., Jin J.L., Yun-Choi H.S., Lee J.R. (2007). Insulin-Mimetic and Insulin-Sensitizing Activities of a Pentacyclic Triterpenoid Insulin Receptor Activator. Biochem. J..

[40] Musto G., Laurenzi V., Annunziata G., Novellino E., Stornaiuolo M. (2022). Genotoxic Assessment of Nutraceuticals Obtained from Agricultural Biowaste: Where Do We “AMES”?. Antioxidants.

[41] Colasante C., Brouard M.O., Pécot-Dechavassine M. (1993). Synaptophysin (P38) Immunolabelling at the Mouse Neuromuscular Junction. Neuromuscul. Disord..

[42] Pratt S.J.P., Valencia A.P., Le G.K., Shah S.B., Lovering R.M. (2015). Pre- and Postsynaptic Changes in the Neuromuscular Junction in Dystrophic Mice. Front. Physiol..

[43] Liu Z., Jin Y.-Q., Chen L., Wang Y., Yang X., Cheng J., Wu W., Qi Z., Shen Z. (2015). Specific Marker Expression and Cell State of Schwann Cells during Culture In Vitro. PLoS ONE.

[44] Jessen K.R., Brennan A., Morgan L., Mirsky R., Kent A., Hashimoto Y., Gavrilovic J. (1994). The Schwann Cell Precursor and Its Fate: A Study of Cell Death and Differentiation during Gliogenesis in Rat Embryonic Nerves. Neuron.

[45] Friedrich R.P., Schlierf B., Tamm E.R., Bösl M.R., Wegner M. (2005). The Class III POU Domain Protein Brn-1 Can Fully Replace the Related Oct-6 during Schwann Cell Development and Myelination. Mol. Cell. Biol..

[46] Wakatsuki S., Yumoto N., Komatsu K., Araki T., Sehara-Fujisawa A. (2009). Roles of Meltrin-β/ADAM19 in Progression of Schwann Cell Differentiation and Myelination during Sciatic Nerve Regeneration. J. Biol. Chem..

[47] Prasanth Kumar P.K., Gopala Krishna A.G. (2014). Impact of Different Deacidification Methods on Quality Characteristics and Composition of Olein and Stearin in Crude Red Palm Oil. J. Oleo Sci..

[48] Nappi A., Murolo M., Cicatiello A.G., Sagliocchi S., Di Cicco E., Raia M., Stornaiuolo M., Dentice M., Miro C. (2022). Thyroid Hormone Receptor Isoforms Alpha and Beta Play Convergent Roles in Muscle Physiology and Metabolic Regulation. Metabolites.

[49] Livak K.J., Schmittgen T.D. (2001). Analysis of Relative Gene Expression Data Using Real-Time Quantitative PCR and the 2^−ΔΔCT^ Method. Methods.

[50] Bentzinger C.F., Lin S., Romanino K., Castets P., Guridi M., Summermatter S., Handschin C., Tintignac L.A., Hall M.N., Rüegg M.A. (2013). Differential Response of Skeletal Muscles to mTORC1 Signaling during Atrophy and Hypertrophy. Skelet. Muscle.

[51] Wang C., Yue F., Kuang S. (2017). Muscle Histology Characterization Using H&E Staining and Muscle Fiber Type Classification Using Immunofluorescence Staining. Bio Protoc.

[52] Dubowitz V. (1974). Muscle Biopsy—Technical and Diagnostic Aspects. Ann. Clin. Res..

[53] Miro C., Nappi A., Sagliocchi S., Di Cicco E., Murolo M., Torabinejad S., Acampora L., Pastore A., Luciano P., La Civita E. (2023). Thyroid Hormone Regulates the Lipid Content of Muscle Fibers, Thus Affecting Physical Exercise Performance. Int. J. Mol. Sci..

