# Supplementary material for: Therapeutic Effect of an Ursolic Acid-Based Nutraceutical on Neuronal Regeneration after Sciatic Nerve Injury
Source: Int J Mol Sci. 2024 Jan 11;25(2):902. doi: 10.3390/ijms25020902 (PMC10815361; doi:10.3390/ijms25020902)
Supplement: Supplementary file 1 [file ijms-25-00902-s001.zip › ijms-2784525-supplementary.pdf]

Table S1. List of primers used for real-time PCR.

Gene	**Forward primer (5' → 3')**	**Reverse primer (5' → 3')**
Atrogin-1	CCTGCATGTGCTCAGTGAGGA	CTTCTTGGGTAACATCGTACAAGC
Murf-1	ACCTGCTGGTGGAAAACATC	CTTCGTGTTCCTTGCACATC
Mbp	TCACACACGAGAACTACCCA	CTTGGGATGGAGGTGGTGTT
Oct-6	CTCCTGGGGTCCTTCTAACT	TTATACACAGATGCGGCTCTC
Mpz	TCTCAGGTCACGCTCTATGTC	GCCAGCAGTACCGAATCAG
Sox-10	AGATCCAGTTCCGTGTCAATAA	GCGAGAAGAAGGCTAGGTG
CypA	CGCCACTGTCGCTTTTCG	AACTTTGTCTGCAAACAGCTC

Table S2. Reagents used for immunofluorescence analysis.

**REAGENT**	**SOURCE**	**IDENTIFIER**
Alpha-Bungarotoxin Conjugates	Invitrogen™	B-13422
Anti-Synaptophysin antibody	Abcam	Ab-14692

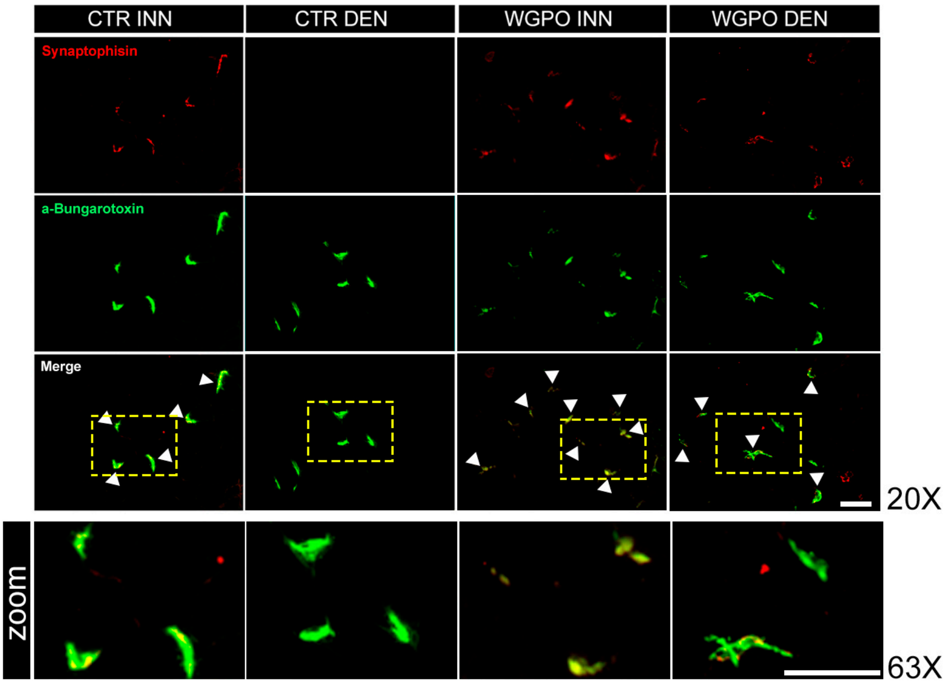

Figure S1. Immunofluorescence staining for α–bungarotoxin and synaptophysin in denervated tibial anterior (TA) muscles compared to innervated muscles of WGPO–pretreated and control (CTR) mice at 10 days after denervation. Scale bar 20X, 50mm. The yellow dashed rectangles highlight the magni-fication area. Magnification 63X, scale bar 50mm. The white arrows in the merged panels indicate the loci of α–bungarotoxin and synaptophysin colocalization.
